# Comparative characterization of inflammatory profile and oral microbiome according to an inflammation-based risk score in ST-segment elevation myocardial infarction

**DOI:** 10.3389/fcimb.2023.1095380

**Published:** 2023-02-13

**Authors:** Paulina Hernández-Ruiz, Luis M. Amezcua-Guerra, Yolanda López-Vidal, Héctor González-Pacheco, Sandra Pinto-Cardoso, Amedeo Amedei, María Magdalena Aguirre-García

**Affiliations:** ^1^ Unidad de Investigación UNAM-INC, División de Investigación, Facultad de Medicina, Universidad Nacional Autónoma de México. Instituto Nacional de Cardiología Ignacio Chávez, Ciudad de Mexico, Mexico; ^2^ Departamento de Inmunología, Instituto Nacional de Cardiología Ignacio Chávez, Ciudad de Mexico, Mexico; ^3^ Programa de Inmunología Molecular Microbiana, Departamento de Microbiología y Parasitología, División de Investigación, Facultad de Medicina, Universidad Nacional Autónoma de México, Ciudad de Mexico, Mexico; ^4^ Unidad de Cuidados Coronarios, Instituto Nacional de Cardiología Ignacio Chávez, Ciudad de Mexico, Mexico; ^5^ Instituto Nacional de Enfermedades Respiratorias Ismael Cosío Villegas, Centro de Investigación en Enfermedades Infecciosas, Ciudad de Mexico, Mexico; ^6^ Department of Experimental and Clinical Medicine, University of Florence, Florence, Italy; ^7^ Interdisciplinary Internal Medicine Unit, Careggi University Hospital, Florence, Italy

**Keywords:** oral microbiome, inflammatory markers, STEMI, cardiac risk, cytokines

## Abstract

Ischemic heart disease considers the myocardial infarction (MI), either non-ST-segment elevation (non-STEMI) or ST-segment elevation myocardial infarction (STEMI); this represents the main cause of mortality in Mexican population. Regarding to the inflammatory state, this is reported to be a major prognostic factor of mortality for patients with MI. One of the conditions capable of producing systemic inflammation is periodontal disease. It has been proposed that the oral microbiota is translocated through the bloodstream to the liver and intestine, generating intestinal dysbiosis. The aim of this protocol is to assess oral microbiota diversity and circulating inflammatory profile in STEMI patients stratified according to an inflammation-based risk scoring system. We found that *Bacteriodetes* phylum was the most abundant in STEMI patients, and *Prevotella* was the most abundant genus, with a higher proportion in periodontitis patients. In fact, *Prevotella* genus was found to correlate positively and significantly with elevated IL-6 concentration. Our study defined a non-causal association inferred between the cardiovascular risk of STEMI patients, determined by changes in the oral microbiota that influence the development of periodontal disease and its relationship with the exacerbation of the systemic inflammatory response.

## 1 Introduction

It is remarkable that in Mexico, ischemic heart disease has regained first place as a cause of mortality in 2021, after COVID-19 (INEGI, 2021). In detail, the ischemic heart disease includes acute myocardial infarction (MI), either non-ST-segment elevation (non-STEMI) or ST-segment elevation MI (STEMI) (Thygesen et al., 2018). The latter is related to atherosclerosis, plaque rupture, and thrombi, an event associated with the highest mortality rate (Crea and Libby, 2017; Anderson and Morrow, 2017; Ridker et al., 2018; Fanola et al., 2017). In acute MI, the systemic inflammatory response is linked with the risk of major adverse cardiovascular events (MACE). This association has been determined by the evaluation of defined inflammatory markers, including high-sensitivity C-reactive protein (hs-CRP), white blood cell count, and albumin. From these markers is obtained the inflammation-based risk scoring system that defines four mortality risk categories: no risk, mild, moderate, and severe, the latter being the one with the highest hospital mortality rate (González-Pacheco et al., 2019). Likewise, other classifications, such as Killip Kimball, TIMI, or GRACE, allow risk stratification of patients with MI (Hung et al., 2021; Williams et al., 2018). Although different causes have been attributed, notably the periodontal disease is a risk factor for various diseases, especially the ischemic heart disease, which was the leading cause of mortality (~16%) until 2019 (WHO, 2021).

According to the World Health Organization, oral diseases affect 3.5 billion people worldwide. Among these conditions is the periodontitis, which is estimated to have a prevalence of 14% in adults (GBD, 2019), while it occupies the tenth cause of morbidity in Mexico (SUIVE, 2020). In this fact, the periodontal disease is a progressive chronic infection in the supporting tissues of the teeth. Depending on the progression degree, it is classified as gingivitis or periodontitis, and many clinical signs are shown, such as gum erythema or bleeding, loss of alveolar bone, and tooth mobility or loss in consequence. (Caton et al., 2018; Tonetti et al., 2018; Kongstad et al., 2017). Various studies have described the microbiota associated with the development of periodontal disease, highlighting the greater abundance of some genera such as *Selenomonas, Dialister, Desulfobulbus*, and *Porphyromonas*, while genera such as *Veillonella, Corynebacterium, Neisseria*, and *Lautropia* are associated with oral health status (Cai et al., 2021). Similarly, studies of different cohorts have identified the relationship between genders such as *Prevotella, Veillonella*, and *Streptococcus* with a higher risk of cardiovascular disease (Fei et al., 2019).

In addition, *Porphyromonas gingivalis* and *Aggregatibacter actinomycetemcomitans*, among other oral cavity pathogens, are capable of translocating into the bloodstream and lodge in atherosclerotic plaques (Figuero et al., 2011). In MI patients, microorganisms belonging to the oral microbiota, such as *Lactobacillus, Bacteroidetes*, and *Streptococcus*, have been isolated from the bloodstream. Likewise, a high proportion of the phyla *Proteobacteria* and *Bacteroidetes* is found in thrombus samples (Zhou et al., 2018; Koren et al., 2011; Kwun et al., 2020). Increasing studies evaluated the interplay between the microbiota of the oral cavity and the intestine. In one study, the genera *Veillonella* and *Streptococcus* were identified in samples analyzed from three different niches, oral cavity, intestine, and atherosclerotic plaque from the same patient (Koren et al., 2011). Likewise, in STEMI patients, alterations of the gut microbiota (GM) have been described, characterized by an increase in *Bacteroides* and *Prevotella*, as well as changes related to a greater GM diversity associated with clinical characteristics of poor oral health (Kwun et al., 2020; Xu et al., 2021; Yin et al., 2015; Gao et al., 2020).

Starting from this scenario, in this study we assessed and correlated the oral microbiota diversity, circulating inflammatory profile in STEMI patients stratified according to cardiovascular risk.

## 2 Article types

Original research articles

## 3 Methods

### 3.1 Study subjects

An observational and cross-sectional study was carried out. Patients were recruited between March 2019 and January 2020, upon admission to the Coronary Care Unit of the National Institute of Cardiology in Mexico City, Mexico (Instituto Nacional de Cardiología “Ignacio Chávez”), an academic tertiary care center dedicated to cardiovascular disease and related conditions. The STEMI diagnosis was based on the findings of the electrocardiogram and the analysis of myocardial lysis markers, following the guidelines of the universal MI definition (Thygesen et al., 2018). Only patients with a first STEMI event of fewer than 72 hours of evolution, who had not received reperfusion therapy or thrombolytic treatment, were included. Patients who did not undergo cardiac catheterization were excluded.

The inflammation-based risk scoring system is a tool originally developed and validated cohort to predict mortality in the context of the acute coronary syndrome, which assesses the state of systemic inflammation. In its evaluation, the serum levels of albumin and hs-CRP are considered, as well as the white blood cells count, according to different pre-established cut-off points that allow the degree of inflammation to be stratified into categories as follows (González-Pacheco et al., 2019): 1 point for counting of white blood cells ≥9.3x10^3^ cells/μl, 2 points for hs-CRP level ≥13.0 mg/l and 3 points for serum albumin level ≥3.6 g/dl. Finally, four categories of systemic inflammation are created based on the total score: 0 points, no signs of systemic inflammation; 1–2 points, mild swelling; 3–4 points, moderate swelling; and 5-6 points, severe inflammation. The serum albumin (reference range 3.5 to 5.7 g/dL) and hs-CRP (reference range <5 mg/L) are photometrically dosed using the AU680 clinical chemistry analyzer (Beckman Coulter, Fullerton, CA), while leukocytes are quantified using the DxH900 automated hematology analyzer (Beckman Coulter, Miami, FL).

### 3.2 Assessment of periodontal disease

To determine the degree of periodontal disease, the following parameters were considered: the presence of dental bacterial plaque or calculus, bleeding and gingival erythema, clinical observation of loss of alveolar crest, tooth mobility, insertion level of the gingival margin at the cement-enamel junction >3 mm, missing teeth and identification of a molar incisor pattern (Kongstad et al., 2017). The oral evaluation was performed by a general dentist, twenty-four hours after admission to the Coronary Care Unit.

### 3.3 Biological samples

Upon patient admission, blood samples were collected in polyethylene terephthalate tubes (BD Vacutainer) with EDTA for plasma and with polymer gel for serum, then obtained by centrifugation and immediately stored at -80°C. Twenty-four hours after hospital admission, healthcare professionals administered a general health questionnaire. Supragingival dental plaque samples were collected by scraping the vestibular and lingual surfaces of all dental organs with a sterilized ¾ Gracey curette. The samples were transferred to a polypropylene sterile container (Eppendorf tube) with 70% ethanol for sample preservation and stored at -20°C until DNA extraction.

### 3.4 DNA extraction and 16s rRNA sequencing

DNA extraction was conducted in-house at Unidad de Investigación UNAM-INC, Instituto Nacional de Cardiología, “Ignacio Chávez”, Mexico City. 16S sequencing was performed by Novogene (China, https://en.novogene.com). The DNA extraction from supragingival plaque samples was performed using the EZ-10 Spin Column Genomic DNA Minipreps kit, Animal (Bio Basic Inc) according to the manufacturer´s instructions. DNA concentration, purity and integrity were verified by spectrophotometer (Nanodrop 1000, ThermoFisher) and gel electrophoresis (ChemiDoc Image System, BioRad) to meet Novogen´s sample submission requirements (available on the company´s website). Extracted DNAs were sent to Novogene for 16s rRNA sequencing. 16s libraries and sequencing were performed according to the Novogene amplicon metagenomics sequencing protocols using the NovaSeq 6000 PE250 platform (Illumina). The amplified 16s rRNA region was V3-V4. Primers and sequences used for amplification were: 341F (F:forward): 5’CCTAYGGGRBGCASCAG-3´ and 806R (R:reverse): 5’GGACTACNNGGGTATCTAAT-3’.

### 3.5 16S data analysis

Demultiplexed raw FastQ files (R1 and R2) were processed using “Quantitative Insights into Microbial Ecology 2” (QIIME2) [v.2020.11] (Bolyen et al., 2019). DADA2 plugin was used to merge paired-end fastQ files, denoised by removing chimeras and constructing a table of amplicon sequence variants (Callahan et al., 2016). Taxonomy was assigned using the Human Oral Microbiome (eHOMD) v.15.2 at 99% identity pre-trained for the V3-V4 region (Escapa et al., 2018). Rarefaction was performed at a sampling depth of 50,000 sequences per sample. Alpha-diversity was estimated using 2 metrics: Shannon and Chao1. Beta-diversity was performed using the Bray Curtis dissimilarity index and visualized using principal coordinate analysis (PCoA). Permutational Multivariate Analysis of Variance (PERMANOVA, adonis, R, 999 permutations) was used to test whether differences between groups. Homogeneity of dispersion was tested using betadisper function (package ‘vegan’ [v.2.6-2], RStudio). Linear discriminant analysis (LDA) effect size (LEfSe) was performed using default parameters and an LDA score equal to or above 3 (Galaxy[v. 1.0]) (Segata et al., 2011).

### 3.6 Cytokine quantification

A panel of 13 cytokines was quantified in serum samples according to the manufacturer´s instructions (LEGENDplexTM Human Essential Immune Response Panel kit, 13plex, Biolegend). The panel included interleukin-4 (IL-4), IL-2, CXCL10 (IP-10), IL-1β, tumor necrosis factor (TNF), CCL2 (MCP-1), IL-17A, IL-6, IL-10, interferon-γ (IFN-γ), IL-12p70, chemokine ligand 8 (CXCL-8), and transforming growth factor-β1 (TGF-β1). Data were obtained on a FACSAria flow cytometer (Biosciences, San Jose, CA, USA) and analyzed with the LEGENDplex™ Data Analysis Software Suite (https://legendplex.qognit.com). As described below, nine cytokines from the panel were selected for this protocol. The assay was performed in triplicate and the reference concentration of each cytokine is presented in **Supplementary Table 1**.

### 3.7 Statistical analysis

Statistical analysis was performed using Fisher exact test for non-continuous variables or Kruskal-Wallis for continuous variables after analyzing the distribution of data using the Shapiro-Wilk test. Correlation analysis was performed by Spearman’s rho, while the calculation of the interquartile range was performed using Tukey’s Hinges.

All analyzes were two-tailed and a p ≤0.05 value was set for significance. For statistical analysis and graphic representation, packages ‘ampvis2’ [v.2.7.23], ‘corrplot’ [v.0.92] and, ‘ggplot2’ [v.3.3.6] of RStudio [v.4.1.2] were used.

## 4 Results

We enrolled twenty-two patients that were classified according to an inflammation-based risk scoring system: no risk (n=3), mild (n=8), moderate (n=7), and severe (n=4). Demographic and clinical characteristics are summarized in the **Table 1**. Significant differences in albumin and hs-CRP concentration were observed in the inflammation-based risk scoring system comparison. It should be noted that all patients had some degree of periodontal disease. Therefore, it was decided to consider the degree of periodontal disease together with the inflammation-based risk scoring system for the metagenomic analysis. We should state that the comparative analysis performed by each group did not show statistical differences between each group.

**Table 1:** Summary of cohort characteristics.

	Without N=3	Mild N=8	Moderate N=7	Severe N=4	p value	
Age, years ^a^	61 (65-58)	60 (54-69)	55 (36-74)	62 (55-70)	0.367	◊
Men ^b^	3(100)	7 (87.5)	7 (100)	3 (75)	0.758	□
Periodontal condition						
Gingivitis ^a^	2 (66)	2 (25)	5 (71.4)	1 (25)	0.272	□
Periodontitis ^a^	1 (33)	6 (75)	2 (28.6)	3 (75)		
Patient medical history						
Diabetes ^a^	1(33)	1 (12.7)	1 (14.3)	2 (50)	0.442	□
Hypertension ^a^	2 (66)	1 (12.7)	2 (28.6)	2 (50)	0.291	□
Dyslipidemia ^a^	1 (33)	Non	3 (42.9)	Non	0.088	□
Current smoking ^a^	3(100)	2 (25)	4 (57.1)	Non	0.037*	□
Previous treatment						
Statin ^a^	Non	2 (25)	1 (14.3)	Non	0.855	□
Aspirin ^a^	Non	2 (25)	1(14.3)	Non	1.000	□
Antihypertensive ^a^	1 (33)	2 (25)	2 (28.6)	1 (25)	1.000	□
Hypoglycemiant ^a^	Non	1 (12.7)	1 (14.3)	1 (25)	1.000	□
Clinical features						
Killip class ≥2 ^a^	Non	3 (37.5)	Non	1 (25)	0.336	□
Affected blood vessel ≥2 ^a^	1(3)	4 (50)	4 (57.1)	3 (75)	0.706	□
TIMI ≥2 ^a^	3 (100)	6 (75)	4 (66.7)	3 (75)	0.901	□
GRACE score ^b^	93 (27)	132 (30)	89 (38)	124.5 (51.5)	0.390	◊
MACE ^b^	1 (33)	1 (12.7)	2 (28.6)	1 (25)	0.821	□
WBC (x10^3^/μL) ^b^	7.7 (0.85)	12.9 (4.1)	10.8 (0.7)	10.1 (14)	0.086	◊
Albumin (g/dL) ^b^	4.0 (0.2)	3.9 (0.3)	3.5 (0.5)	2.9 (0.6)	0.001*	◊
C-reactive prot (mg/L) ^b^	3.1 (2.7)	1.5 (4)	8.7 (12.2)	78.5 (38)	0.016*	◊
cTnI (ng/mL) ^b^	3.9 (9.5)	3.5 (48.9)	0.19 (1.28)	11.6 (42.2)	0.532	◊
Total cholesterol (mg/dL) ^b^	135.6 (20.3)	185.7 (80.1)	171.3 (54.5)	146.5 (66.3)	0.174	◊
HDL (mg/dL) ^b^	37.4 (13.4)	35.9 (10.6)	32.4 (15.9)	27.5 (18.6)	0.705	◊
LDL (mg/dL) ^b^	90.4 (7.7)	125.8 (72.7)	108.4 (54.9)	91 (48.7)	0.178	◊
BMI ^b^	26.4 (1.6)	26.8 (4.8)	28.1 (1.1)	28.2 (8.8)	0.690	◊

^a^Frequency (%), ^b^Median (IQR), p value ◊: Kruskal-Wallis or □: Fisher exact test. MACE, mayor adverse cardiovascular event; WBM, white blood monocytes; C-reactive prot, C-reactive protein; cTnI, Cardiac troponin I; HDL, high density protein; LDL, low density protein; BMI, body mass index.

### 4.1 Variance of oral microbiota diversity associated with risk classification

The analysis of the oral microbiota was established by identifying the variance of the samples through the beta diversity by Bray Curtis index according to the different risk stratification parameters: inflammation-based risk scoring system, Killip Kimball class, number of affected blood vessels, TIMI and GRACE scores, and presence of MACE. The **Supplementary Table 2** defines the estimated risk ranking parameters for the beta diversity analysis. The homogeneity analysis of the dispersion of groups was assigned for the inflammation-based risk scoring system and the degree of periodontal disease, where homogeneity between the groups was demonstrated (p<0.05, BETADISPER).

**Figure 1:**
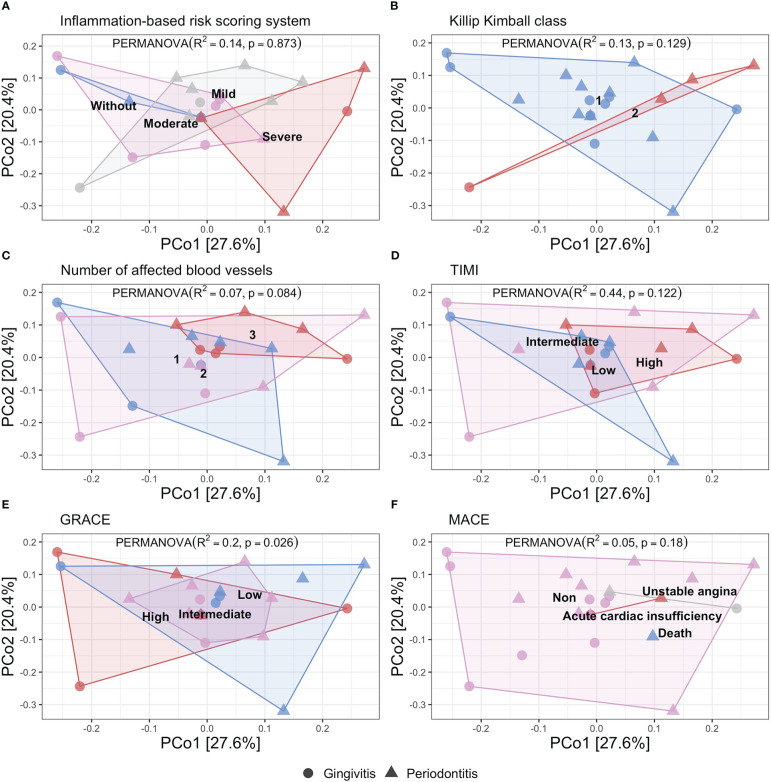
Principal coordinate analysis of beta diversity by Bray Curtis index related to different risk score in STEMI patients showed by periodontal disease. (PERMANOVA, R2, p value). **(A)** Inflammation-based risk scoring system: Without risk (blue), Mild risk (gray), Moderate risk (pink), and, Severe risk (red) (R^2^ = 0.14, p=0.873). **(B)** Killip Kimball class 1: patients without clinical signs of heart failure (blue), class 2: mild heart failure (red) (R^2^ = 0.13, p=0.129). **(C)** Number of affected blood vessels: one (blue), two (pink), and three (red) blood vessels affected (R^2^ = 0.07, p=0.084). **(D)** Thrombolysis in myocardial infarction score (TIMI): low (blue), intermediate (pink), high (red) (R^2^ = 0.44, p=0.122). **(E)** GRACE score: low (blue), intermediate (pink), high (red) (R^2^ = 0.20, p=0.026). **(F)** Mayor adverse cardiovascular event (MACE): unstable angina (gray), acute coronary insufficiency (red), death (blue), non-MACE (pink) (R^2^ = 0.05, p=0.18).

In the **Figure 1** we show the principal coordinate analysis determining the distribution of the patients according to the inflammation-based risk scoring system, considering that 14% of the sample variance is related to the categories of this classification (R^2^ = 0.14, p=0.873). The Killip Kimball class shows that most of the patients were classified without heart failure and it is determined that 13% of the variance is related to the degree of heart failure (R^2^ = 0.13, p=0.129). The number of affected blood vessels is defined by the involvement of the coronary arteries: anterior descending, circumflex, and right coronary; this variable presented a variance of 7% (R^2^ = 0.07, p=0.84). TIMI and GRACE scores determine a variance of 44% and 20%, respectively, resulting in a significant difference for the GRACE score (R^2^ = 0.44, p=0.122; R^2^ = 0.20, p=0.026, respectively). Finally, the presence of MACE had a variance of 5% (R^2^ = 0.05, p=0.8).

According to the variance analysis for beta diversity described in the different included scores, we found that the cardiovascular risk was not influenced by the oral microbiome diversity, although we observed the different distribution for the gingivitis and periodontitis patients (the last mentioned, distributed in high-risk category in different scores). Particularly, in the inflammation-based risk scoring system, we observed the major proportion of periodontitis patients. Instead, we performed the same variance analysis of beta diversity according to the degree of periodontal disease, we showed the patients’ distribution according to the degree of periodontal disease, considering that 3.9% of the variance of the sample is related to the categories of this classification (R^2^ = 0.039, p=0.455) (**Supplementary Figure 1A**). We documented that the oral microbiota diversity was dependent on each patient; instead, we performed the metagenomic analysis looking for a different pattern in each classification of the inflammation-based risk scoring system.

### 4.2 Metagenomic analysis

**Figure 2:**
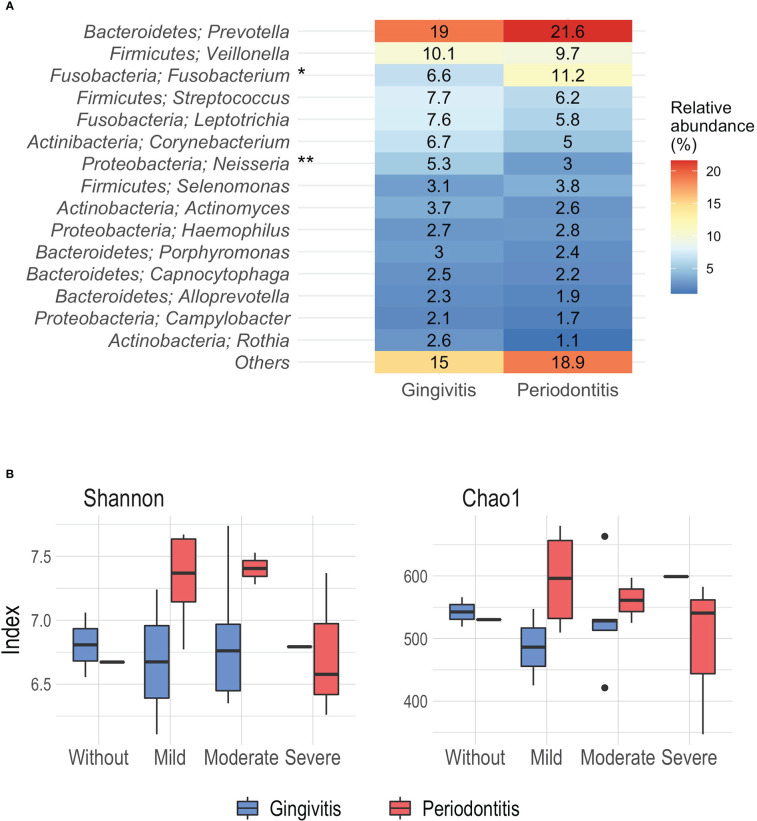
**(A)** Relative abundance (%) of the main phyla and genera associated to periodontal disease. **(B)** Alpha diversity analysis by Shannon and Chao1 index according the inflammation-based risk scoring system and periodontal disease. *p= 0.008, **p=0.03, Mann-Whitney.

In general, in the STEMI patients, we observed that the phyla *Bacteroidetes, Firmicutes, Fusobacteria, Actinobacteria*, and *Proteobacteria* represented 97% of the proportion of the samples and the most abundant was *Bacteroidetes*. As previously reported, this analysis was performed with patients grouped by inflammation-based risk scoring system and degree of periodontal disease. Regarding the relative abundance of the main types of genera according to the degree of periodontal disease, we documented that *Prevotella* was the most abundant genus, with a higher proportion in periodontitis’ patients, followed by *Fusobacterium* and *Veillonella*; while, in patients with gingivitis, *Prevotella, Veillonella*, and *Streptococcus* were present in greater abundance. Similarly, differences were identified in genera such as *Fusobacterium* (p=0.008) and *Neisseria* (p=0.03) between the degree of periodontal disease, the latter being observed more frequently in gingivitis patients (**Figure 2A**).

Likewise, the significance analysis of the alpha diversity was carried out considering the degree of periodontal disease and the inflammation-based risk scoring system as grouping variables. At this point, no significant differences were reported. Using the Shannon index, a greater diversity was observed in patients with periodontitis classified as moderate and mild risk (median 7.4 and 7.36); while, the lowest index of diversity was noted in periodontitis patients with severe risk (median 6.57) (**Figure 2B**). On the other hand, the analysis derived from the Chao1 index showed that the highest richness was identified in periodontitis patients mild risk (mean 596.11), and the lowest richness was observed in patients with gingivitis and mild risk (mean 486.25) (**Figure 2B**).

In order to identify differential taxa between the groups in relation to the degree of periodontal disease, LEfSe analysis (linear discriminant analysis with effect size) was performed. We documented that some genera such as *Fusobacterium* and *Atopobium* were found to be associated with periodontitis patients, while in patients with gingivitis the association between *Lautropia* and *Neisseria* was observed (LDA score 3.0, p=0.05) (**Supplementary Figure 1B**). In relation to the grouping of inflammation-based risk scoring system, it was not possible to establish differences using the latter analysis. As shown, there was a linkage between *Prevotella* abundance in the STEMI patients, further, we observed differences according to the abundance of genus related to the degree of periodontal disease, suggesting that the oral microbiome diversity of the STEMI patients, had related to the oral health status; hence, the significant differences found, inferred in a modification of the oral microbiome diversity, related to the degree of the periodontal disease.

**Figure 3:**
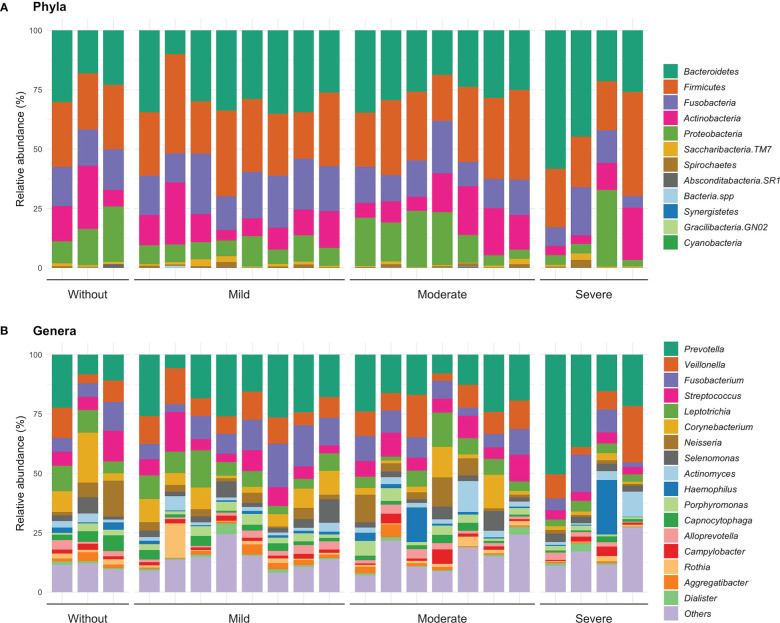
Relative abundance at phyla **(A)** and genera level **(B)** according the inflammation-based risk scoring system. In this same figure, the category of “others” is identified, where the identified genders are grouped in a proportion of less than 1%.

Regarding the description of patients with gingivitis and severe risk, the *Bacteroidetes* was the most frequent, while in the other risk groups, *Firmicutes* was the most abundant. On the other hand, in patients with periodontitis and without or moderate risk, the phylum *Firmicutes* showed a greater abundance, as well as in patients with periodontitis and mild and severe risk, *Bacteroidetes* was the most abundant (**Figure 3A**).

About genus distribution, we observed that *Prevotella* presented a greater abundance in most of the levels of the inflammation-based risk scoring system. In detail, patients with gingivitis and no risk had a greater abundance of *Corynebacterium* (14.8%) and *Leptotrichia* (10.1%); patients with mild risk had a higher proportion of the genera *Veillonella* (13.3%) and *Streptococcus* (11.7%); while, in patients with moderate and severe risk, *Veillonella* (9.6 and 10.1%) and *Fusobacterium* (7.8 and 4.9%) were more frequent. Differences were observed between gingivitis and periodontitis in patients with mild risk in genera such as *Fusobacterium* (p=0.046) and *Aggregatibacter* (p=0.046); while in patients with moderate risk, a trend was identified in genera such as *Neisseria* (p=0.053), *Haemophilus* (p=0.053) and *Capnocytophaga* (p=0.053) (**Figure 3B**). These observations suggest a linkage between specific genus related to a high or low risk identified by the inflammation-based risk scoring system, signifying that the named genus of the oral microbiota may be analyzed further like a risk marker in STEMI patients. Hence, according to the inflammation-based risk scoring system, the severe risk patients showed a great abundance of periodontal pathogens, mainly gram-negative bacteria; this observation may suggest the linkage between the degree of periodontal disease and an exacerbation of the inflammatory profile in the STEMI patients.

### 4.3 Serum cytokines profiling

**Figure 4:**
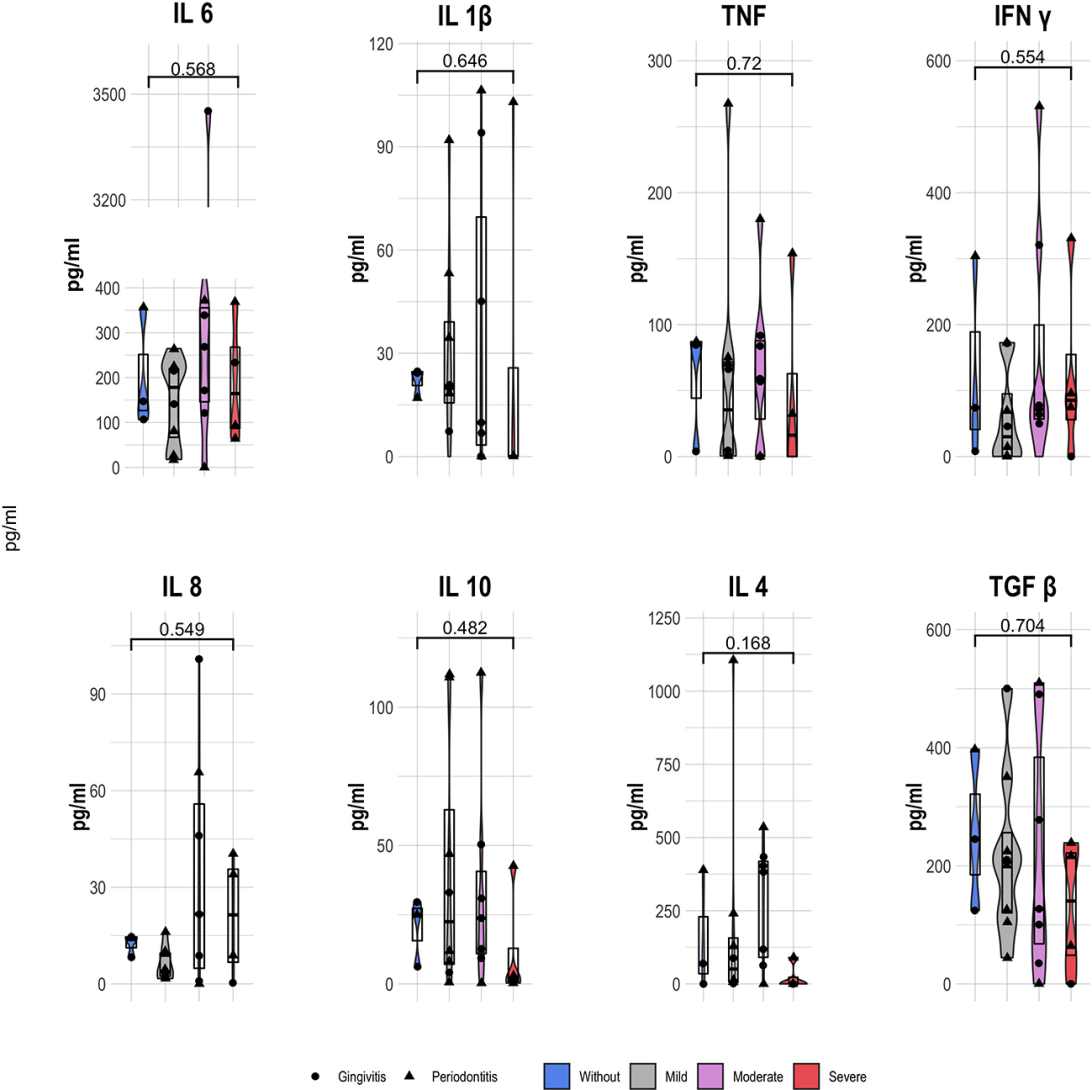
Serum cytokine concentration (pg/ml) of STEMI patients according the inflammation-based risk scoring system; showing the median and 25th y 75th quartile; p value, Kruskal Wallis.

We analyzed the cytokine profile in the patients, grouped according to the inflammation-based risk scoring system and considering their distribution respecting the degree of periodontal disease. Regarding the concentration analysis of each group within the inflammation-based risk scoring system, we observed that the patients categorized without risk presented a higher median concentration of IL-1β (24.19 pg/mL), TNF (84.65 pg/mL), IL-10 (25.01 pg/mL), and TGF-β1 (245.31 pg/mL), as well as a IL-6 decrease (146.73 pg/mL) than the other risk groups. In patients with moderate inflammation, we documented a higher concentration of IL-6 (267.83 pg/mL), IL-8 (21.67 pg/mL), IL-4 (382.31 pg/mL), and IL-17A (18.25 pg/mL) than other groups. Finally, in patients with severe risk, we found higher IFN-γ (85.24 pg/mL) and lower levels of IL-1β, TNF, IL-10, IL-4, and IL-17A; while in patients with mild risk, a lower concentration of IFN-γ and IL-8 was observed (**Figure 4**). No significant differences were found in the comparisons between risk groups of the inflammation-based risk scoring system and the cytokines profile.

Finally, we evaluated the relationship of the concentration of each cytokine in STEMI patients, as well as their grouping by means of the inflammation-based risk scoring system. As noted, no significant differences between risk groups were found. We registered a similar situation with no differences, comparing the degree of periodontal disease, but the highest concentration of all cytokines are documented on patients with periodontitis. Similarly, a slightly positive correlation between the inflammation-based risk score and the concentration of IL-6 (p=0.564), IFN-γ(p=0.419), and IL-8(p=0.481) was found; while the degree of periodontal disease presented a slightly anti-correlation with the levels of IL-6 (p=0.369) and a positive correlation with the other cytokines (**Supplementary Table 1**). As shown, the serum cytokines’ profiling did not present significant differences between groups; although we proposed the correlation analysis between oral microbiota genera and the inflammatory profile.

### 4.4 Correlation between the most abundant genera and serum cytokines

**Figure 5:**
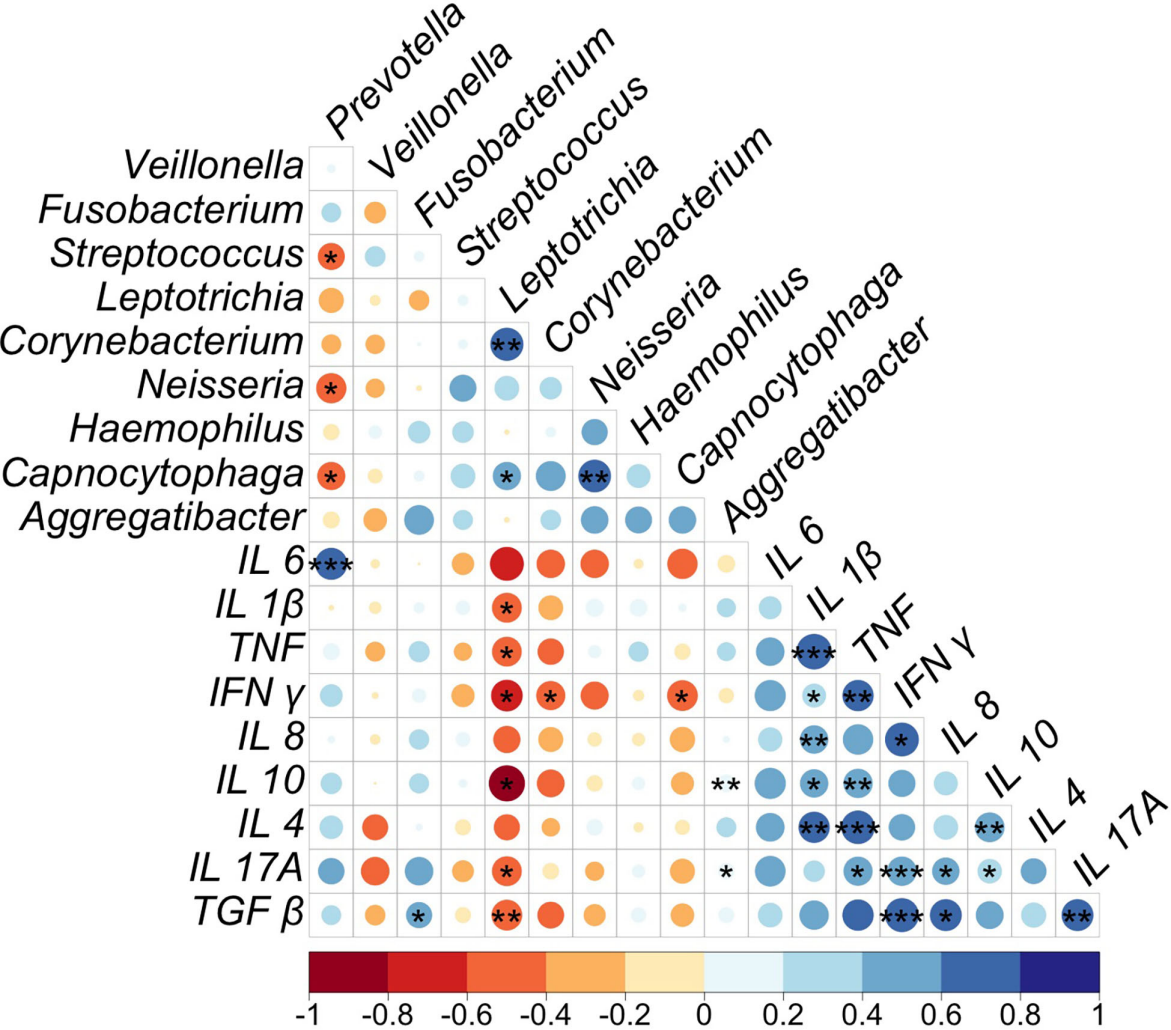
Spearman correlation test of oral microbiota diversity and serum cytokines concentration in STEMI patients; p value: *p ≤ 0.05, **p ≤0.01, ***p ≤ 0.001.

In order to define a potential correlation between the microbiota diversity and the inflammatory profile of the patients, a Spearman correlation analysis was applied. On the **Figure 5** we reported the genus with the greatest abundance, some of them were identified with statistically significant differences comparing different variables, likewise, the different pro and anti-inflammatory cytokines quantified in serum. At first glance, considering all the patients of STEMI cohort, the *Prevotella* genus was found to correlate positively and significantly (p=0.0002) with elevated IL-6 concentration, as well as lower IL-1β concentration. The *Veillonella* genus showed a mild negative correlation with all the analyzed cytokines, as well as a moderate negative correlation with the IL-4 concentration; while *Fusobacterium* presented a positive correlation with the concentration of cytokines, resulting in a significant positive correlation (p=0.05) with TGF-β. The genus *Streptococcus* showed a non-significant positive correlation with the concentration of IL-1β, IL-8 and IL-10. Notably, a greater abundance of genera such as *Leptotrichia* and *Corynebacterium* correlated with a lower concentration of cytokines, observing a considerable and significant anti-correlation between *Leptotrichia* and the concentration of IL -1β (p=0.03), TNF (p=0.02), IFN-γ (p=0.02), IL-10 (p=0.02), IL-17A (p=0.05) and TGF-β (p=0.006), while *Corynebacterium* presented a moderate and significant positive correlation with the IFN-γ levels (p=0.05). On the other hand, *Neisseria* presented a non-significant moderate anti-correlation with IL-6, IFN-γ and TGF-β, while in relation to *Haemophilus*, no important correlations were observed. Finally, *Capnocytophaga* showed a significant and negative correlation with IFN-γ (p=0.05) while *Aggregatibacter* a slight and significant positive correlation with both IL-10 (p=0.005) and IL-17A (p=0.02) (**Figure 5**).

Finally, considering the correlation analysis between the concentration of all the cytokines, we observed a slight to moderate positive correlation between these defined biomarkers with different significance degrees (**Figure 5**). In addition, we identified the interaction between the most abundant bacterial genera, documenting, i) an inverse and significant correlation between *Prevotella* and genera such as *Streptococcus* (p=0.02)*, Neisseria* (p=0.05) and *Capnocytophaga* (p=0.04); ii) a positive and significant correlation between genera such as *Leptotrichia* and *Corynebacterium* (p=0.003) and *Capnocytophaga* (p=0.05); and iii) a moderate and significant positive correlation of this last genus with *Neisseria* (p=0.002). In conclusion, the most relevant statement found in the correlation analysis was the finding of the linkage of *Prevotella* with the IL-6 concentration; this statement emphasizes the role of the gram-negative in the exacerbation of the pro-inflammatory profile in the STEMI patients.

## 5 Discussion

In the present study, analyzing the oral microbiome we documented a relationship of the *Bacteroidetes* phyla in the STEMI development, and especially the genus *Prevotella*, which a greater abundance in patients with severe risk. In detail, we observed a significant decrease in the phylum *Firmicutes* and the genus *Haemophilus*, as well as an increase *Bacteroidetes* phylum compared to healthy controls. In addition, the phylum *Bacteroidetes* was the most abundant mainly in patients with severe risk. Likewise, the analysis carried out in different populations in relation to cardiovascular disease risk factors determined that *Prevotella, Veillonella* and *Streptococcus* are usually found in greater abundance in patients with high cardiovascular risk, while *Neisseria* is associated with a lower risk of cardiovascular disease (Kwun et al., 2020; Fei et al., 2019). Different studies carried out in human MI or atherosclerosis have documented a greater abundance of the phylum *Firmicutes*; however, it must be remarked that the analyzed samples differ from the sampling of our study (Koren et al., 2011; Menon et al., 2017; Fåk et al., 2015).

The oral evaluation of our cohort determined that all patients showed some degree of periodontal disease, mainly periodontitis (in more than half of the population); these patients presented a greater abundance of the genus *Prevotella* and *Fusobacterium* and the latter showed a difference statistically significant compared to gingivitis patients. In agreement with our data, *Fusobacterium* is previously identified in patients with periodontitis in a positive relationship with respect to a more aggressive disease degree. In addition, in a different cohort of periodontitis patients, *Prevotella* was identified as the most abundant genus (Cai et al., 2021; Belstrøm et al., 2021). Likewise, we have observed that the genus *Neisseria* decreased in subjects with periodontitis, in agreement with other studies that documented this genus associated with oral health status. Finally, other groups identified the genera *Veillonella* and *Streptococcus* with greater abundance in healthy people. In the present cohort, both genera were abundantly identified in gingivitis patients. At this point, it should be considered that the gingivitis diagnosis was obtained mainly by erythema and gingival bleeding, probably associated with the inability of patients to perform their oral hygiene, ruling out signs of severe periodontal disease (Cai et al., 2021).

Usually there are different relationships between bacterial microbiota genera. In general, in this study, a negative correlation of *Prevotella* with genera such as *Streptococcus, Neisseria* and *Capnocytophaga* was determined. This finding are in line with the analysis of periodontitis patients, where there is a negative correlation of pathobionts in relation to genera associated with oral health, such as *Capnocythophaga, Rothia, Veillonella, Streptococcus, Haemophilus, Corynebacterium, Leptotrichia* and *Actinomyces* (Cai et al., 2021; Ai et al., 2017; Chen et al., 2018; Marotz et al., 2022). In addition, in a previous study focused on patients who received periodontal dental treatment, the authors observed a decrease in pathogenic genera such as *Porphyromonas, Tanerella, Prevotella* and *Filifactor*, as well as an increase of previously mentioned species associated with health, sustaining that the microbiota is significantly associated with the development of periodontal disease and that this can be modified with dental procedures (Chen et al., 2018). Even in patients with a history of coronary artery disease, including a previous MI, the authors documented a relationship between periodontal therapies and a decrease in the concentration of biomarkers associated with these diseases (Saffi et al., 2013).

Regarding the alpha diversity analysis of this cohort of patients, no statistically significant differences were found comparing the grouping variables, however, it was observed that the abundance and richness decrease in periodontitis patients with severe risk. Comparing periodontitis with healthy controls, some studies documented no significant differences in the species’ abundance but a lower richness in periodontitis patients. On the contrary, other authors observed a greater diversity of species in periodontitis patients, with a diversity decrease in progressive periodontitis. These discrepancies may be probably related to differences in data processing and samples’ collection and also the enrolled population (Cai et al., 2021; Ai et al., 2017; Chen et al., 2018; Plachokova et al., 2021). On the other hand, the alpha diversity identified in the oral cavity of STEMI patients determined a greater diversity than in healthy controls (Kwun et al., 2020). Similarly, the comparison between healthy people and patients with atherosclerotic cardiovascular disease, registered no significant differences in alpha diversity; however, a decrease is observed in patients with heart disease (Kato-Kogoe et al., 2022).

Finally, the beta diversity analysis of this cohort determined that the variance of the sample is not influenced by the periodontal health status, as previously reported in patients with periodontitis, documenting a variance of less than 1% associated with the periodontal status (Belstrøm et al., 2017). However, an association was observed in the greater variance, with respect to cardiovascular risk stratification through the TIMI score; suggesting that the diversity of the oral microbiota influences the greater or lesser cardiovascular risk (Belstrøm et al., 2017).

Regarding the IL-6 concentration in acute coronary syndrome patients, we documented a higher median concentration in our cohort compared with previous studies. At this point, it should be considered that the elevated concentration of IL-6 is related to the MACE development (Fanola et al., 2017; Gao et al., 2020; Ridker et al., 2020). On the other hand, the analysis of a cohort of patients with severe CVD (cardiovascular disease) detailed that the concentration of IL-4 and IL-17 in severe disease decreased comparing patients with mild CVD and non-CVD; suggesting a protective effect of these cytokines (Liu et al., 2022). This same relationship was observed in our cohort of STEMI patients, where a decrease in the concentration of IL-4 and IL-17A is observed in patients with severe risk. These data are apparently contrasting with previous studies reporting that IL-17A levels <6.26 pg/ml in serum are related to a higher risk of post-infarction mortality; at this point, the only death recorded in the present cohort coincided with the highest serum concentration of IL 17A (Simon et al., 2013). Regarding the relationship between the IL-17A concentration and other cytokines, we observed a positive and significant correlation with TNF, IFN-γ and IL-8 in agreement with data registered in obese patients predisposed to atherosclerotic disease (Tarantino et al., 2014).

On the other hand, considering the inflammation-based scoring system, in patients without risk, it was observed that the genus *Corynebacterium* was present in greater abundance in gingivitis patients; this is related to the findings of a different cohort, which determined a negative association of *Corynebacterium matruchotti* with the diagnosis of aggressive periodontitis, as well as an inverse relationship with analyzed markers of inflammation, such as IL-6, IL-1Ra and leukocyte count (Plachokova et al., 2021).

Similarly, the relationship of severe periodontitis in MI patients has been described. In a cohort of 100 patients, a decrease in the plasma TGF-β concentration was observed, as well as a concentration ≥2 mg/L of hs-CRP and, consequently, elevated levels of IL-6 (Lira et al., 2021). Regarding the TGF-β concentration, we observed a decrease in patients with moderate risk, as well as an increased concentration of IL-6 and hs-CRP in periodontitis patients.

The inflammatory profile has also been analyzed in patients with periodontitis, observing that, in general, the cytokines’ concentration is higher in these patients, without statistically significant differences; on the contrary a decrease in cytokines’ levels was documented only after periodontal treatment (Montenegro et al., 2019). It is worth mentioning that the mean IL-10 concentration in this population is above the basal concentration reported in patients with hypercholesterolemia (1.07 ± 0.55 pg/ml), in addition to showing an anti-correlation with LDL levels, highlighting the IL-10 role in an anti-atherogenic process (Barale et al., 2018).

## 6 Conclusion

The importance in the care of ischemic heart diseases in our population implies the analysis of the different risk factors that influence the development of these diseases.

We are aware that our study shows some limitations such as the restricted number of patients and the missing interplay between gut and oral microbiota, but in this analysis, a non-causal association is inferred between the cardiovascular risk of STEMI patients, determined by changes in the oral microbiota that influence the development of periodontal disease and its relationship with the exacerbation of the systemic inflammatory response. Further analysis is necessary, emphasizing the cardiovascular risk markers present in these patients to clarify the fine mechanisms associated with dysbiosis and its influence as a risk factor in the establishment of human STEMI.

## 7 Limitations

This protocol was a pilot study to determine the main differences according the microbiota and biomarkers in STEMI subjects, we expected to enlarge the cohort and analyze a relation between the oral and gut microbiome.

## Data availability statement

The datasets presented in this study can be found in online repositories. The names of the repository/repositories and accession number(s) can be found below: https://www.ncbi.nlm.nih.gov/bioproject/PRJNA878487.

## Ethics statement

The studies involving human participants were reviewed and approved by Comité de Ética en Investigación, Instituto Nacional de Cardiología Ignacio Chávez, protocol number 18-1089. The patients/participants provided their written informed consent to participate in this study.

## Author contributions

MMA-G, HG-P, LA-G, AA conceived and supervised the protocol design, and wrote the manuscript. PH-R, and MMA-G design experiments. PH-R, recruited and collected patient data, obtain tissue samples, performed the oral evaluation, processed the patient samples, performed extraction, and analyzed DNA, chemokine determination, analyzed and discussed data, designed figures and tables, and wrote the manuscript. HG-P and LA-G supervised and contributed to recruitment, diagnosis and analysis of clinical parameters. YL-V and SP-C contributed to 16S rRNA data analysis, discussed data, and wrote manuscript. All authors contributed to the article and approved the submitted version.
