# Supplementary material for: Comparative characterization of inflammatory profile and oral microbiome according to an inflammation-based risk score in ST-segment elevation myocardial infarction
Source: Front Cell Infect Microbiol. 2023 Feb 13;13:1095380. doi: 10.3389/fcimb.2023.1095380 (PMC9968971; doi:10.3389/fcimb.2023.1095380)
Supplement: Supplementary file 1 [file DataSheet_1.docx]

|  | STEMI | Without | Mild | Moderate | Severe | p value | Correlation test  Rho Spearman | |
| --- | --- | --- | --- | --- | --- | --- | --- | --- |
|  |  |  |  |  |  |  | Inflammation-based risk score | Periodontal disease |
| IL 6 | 193.56 (173.23) | 146.73  (173.23) | 178.16  (168.25) | 267.83  (209.58) | 164.40  (222.61) | 0.568 | 0.130 | -0.201 |
| IL 1β | 19.22 (45.22) | 24.19  (45.22) | 20.52  (30.92) | 9.86  (66.26) | 0.00  (51.52) | 0.646 | -0.241 | 0.073 |
| TNF | 57.91 (84.14) | 84.65  (84.14) | 35.40  (71.45) | 58.98  (59.32) | 16.18  (93.20) | 0.720 | -0.194 | 0.072 |
| IFN γ | 70.59 (163.36) | 73.95 (163.36) | 30.02  (119.83) | 71.67  (142.54) | 85.24  (176.15) | 0.554 | 0.182 | 0.036 |
| IL 8 | 9.12 (19.23) | 14.23  (19.23) | 3.87  (7.38) | 21.67  (51.02) | 21.43  (32.62) | 0.549 | 0.158 | 0.058 |
| IL 10 | 18.21 (38.57) | 25.01  (38.57) | 22.46  (72.75) | 23.75  (29.67) | 1.93  (22.16) | 0.482 | -0.198 | 0.029 |
| IL 4 | 78.78 (380.89) | 69.55  (380.89) | 50.83  (177.67) | 382.31  (327.89) | 0.07  (45.20) | 0.168 | -0.130 | 0.014 |
| IL 17A | 13.26  (23.47) | 15.31  (17.85) | 12.77  (18.35) | 18.25  (31.72) | 3.66  (38.67) | 0.624 | -0.081 | 0.043 |
| TGF β | 205.59 (176.75) | 245.31  (176.75) | 205.59  (172.47) | 126.73  (315.99) | 140.39  (195.11) | 0.704 | -0.241 | 0.007 |

**Supplementary Table 1**. Cytokines’ levels of STEMI patients according the inflammation-based risk score; showing the median and interquartile range calculated by Tukey range test. p value, Kruskal Wallis test; 1𝛽 under-power. Correlation test, Spearman (Rho value).

| Risk classification | Scale | Parameters estimated |
| --- | --- | --- |
| Killip Kimball class | 1-4 class | Class 1: patients without clinical signs of heart failure  Class 2: Mild heart failure  Class 3: Pulmonary edema  Class 4: Cardiogenic shock doi.org/10.1016/j.amjcard.2015.01.010 |
| Number of affected blood vessels | 1-3 number | Anterior descending,  Circumflex  Right coronary |
| TIMI | 1-14 points | Low: 0-2  Intermediate: 3-4  High: 5 or more  <https://doi.org/10.1161/JAHA.112.003269> |
| GRACE score | Points | Low: <108  Intermediate: 109-140  High: >140 |

**Supplementary Table 2.** Definition of the risk classification parameters estimated for the beta diversity analysis.

**Supplementary Figure 1**. Flow chart of the study cohort


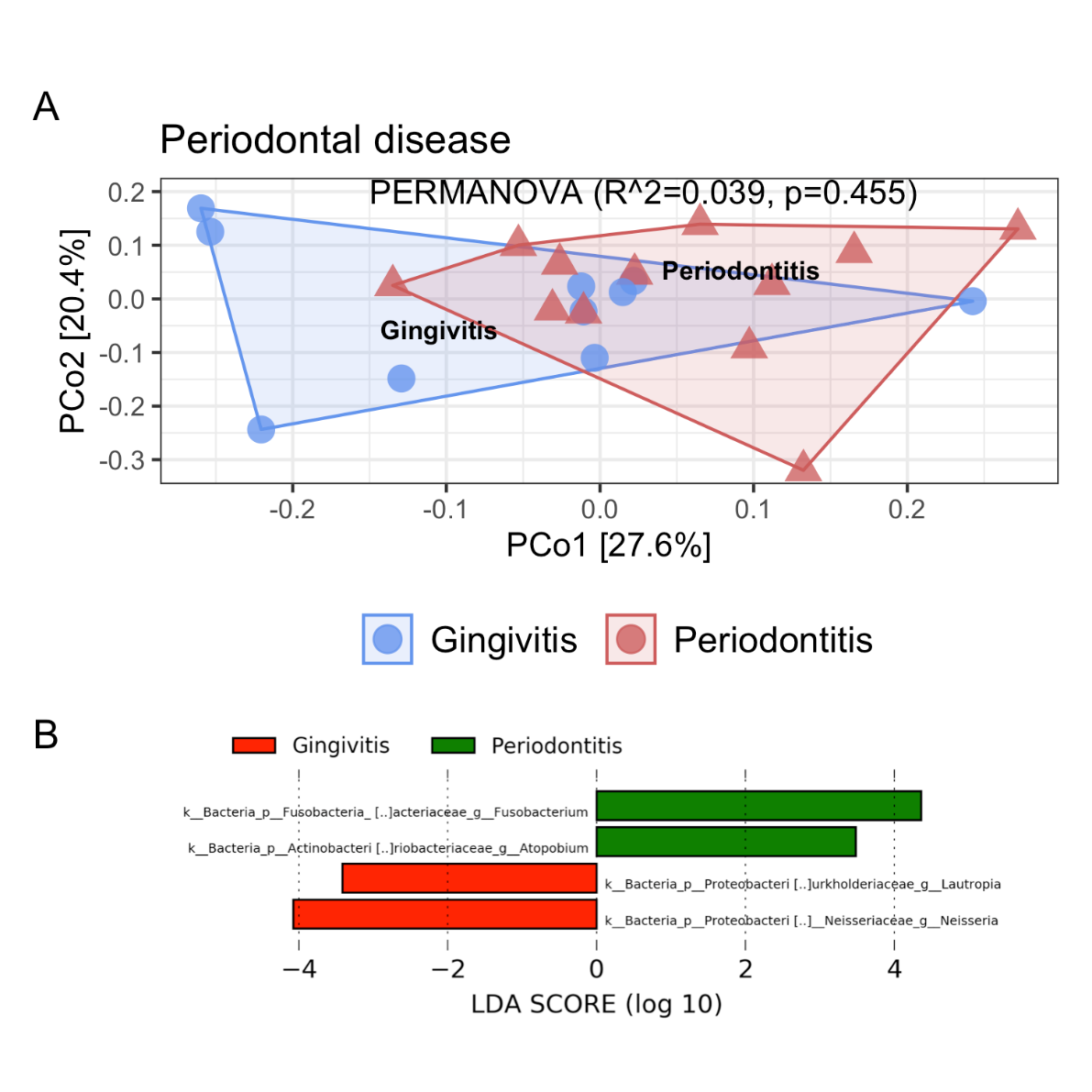


**Supplementary figure 2.** A) Principal coordinate analysis of beta diversity by Bray Curtis index in STEMI patients shown by periodontal disease (PERMANOVA, R^2^=0.039, p= 0.455). B) LDA Effect Size (LEfSe) related to periodontal disease. LDA score 3.0, p=0.05
